# Endocrine and metabolic disorders in patients with Gaucher disease type 1: a review

**DOI:** 10.1186/s13023-019-1211-5

**Published:** 2019-12-02

**Authors:** Małgorzata Kałużna, Isabella Trzeciak, Katarzyna Ziemnicka, Maciej Machaczka, Marek Ruchała

**Affiliations:** 1 Ward of Endocrinology, Metabolism and Internal Diseases Ward, Heliodor Swiecicki University Hospital, Poznan, Poland; 2 Department of Endocrinology Metabolism and Internal Diseases, Poznan University of Medical Sciences, Poznan, Poland; 3 Medical Faculty, University of Rzeszow, Rzeszow, Poland; 4 Department of Clinical Science and Education, Division of Internal Medicine, Södersjukhuset, Karolinska Institutet, Stockholm, Sweden

**Keywords:** Gaucher disease (GD), Obesity, Malnutrition, Insulin resistance, Growth hormone deficiency, Delayed puberty, Leptin, Adpionectin, Dyslipidaemia, Vitamin D deficiency

## Abstract

**Background:**

Gaucher disease (GD) is one of the most prevalent lysosomal storage diseases and is associated with hormonal and metabolic abnormalities, including nutritional status disorders, hypermetabolic state with high resting energy expenditures, peripheral insulin resistance, hypoadiponectinaemia, leptin and ghrelin impairments, hypolipidaemia, linear growth deceleration and growth hormone deficiency, delayed puberty, hypocalcaemia and vitamin D deficiency. Specific treatments for GD such as enzyme replacement therapy and substrate reduction therapy display significant effects on the metabolic profile of GD patients.

**Main body of the abstract:**

Hormonal and metabolic disturbances observed in both adult and paediatric patients with Gaucher disease type 1 (GD1) are discussed in this review. The PubMed database was used to identify articles on endocrine and metabolic disorders in GD1. GD1 appears to facilitate the development of disorders of nutrition, glucose metabolism and vitamin D insufficiency. Metabolic and hormonal diseases may have a significant impact on the course of the underlying disease and patient quality of life.

**Conclusions:**

Conditions relating to hormones and metabolism can be wide-ranging in GD1. Obtained findings were intrinsic to GD either as a deleterious process or a compensatory response and some changes detected may represent co-morbidities. Actively seeking and diagnosing endocrine and metabolic disorders are strongly recommended in GD1 patients to optimize healthcare.

## Background

Gaucher disease (GD) is an autosomal recessive disorder which occurs in approximately 1 in 40,000–50,000 live births [1]. It results from insufficient activity of the enzyme glucocerebrosidase (acidic β-glucosidase) [1, 2]. A very small minority of GD is caused by saposin C deficiency [1, 2]. GD affects various tissues and organs in the body, particularly bone marrow, spleen, liver and lungs. Pathogenic mutations of the *GBA* gene (encodes glucocerebrosidase) located on chromosome 1q21.31or *PSAP* gene (encodes prosaposin) located on chromosome 10q22.1, underlie GD. The progressive accumulation of glucocerebroside causes clinical manifestations of the disease [1–5]. The classification of clinical subtypes 1, 2, and 3 is useful in anticipating the prognosis and establishing the proper genetic counselling alongside management. Primary central nervous system involvement is characteristic of subtypes 2 and 3 [4–6]. Type 2, which is also called acute or infantile, is linked with poor prognosis with most patients dying before the age of 2. GD1 is the most common type of GD [1]. Hence, the current study presents GD type 1 (GD1) hormonal disturbances.

Hormonal and metabolic disturbances in GD1 are clinically accompanied by symptoms that influence a patient’s quality of life (QoL). Hormonal disturbances may substantially affect their general health. Since few studies have been conducted to assess the benefits of ERT on hormonal, nutritional and metabolic disorders, the objective of this study was to review the literature concerning the hormonal and metabolic status of GD1 patients, both on ERT/SRT and without ERT/SRT.

The Pubmed database was searched with the following study parameters: clinical trials, systematic reviews, case reports and meta-analyses. The results of interest were the following: malnutrition, overweight, obesity, glucose metabolism, insulin resistance, diabetes, cholesterol metabolism, disturbances in adiponectin, leptin, and ghrelin levels, linear growth deceleration and growth hormone deficiency, gynaecological symptoms, thyroid diseases, endocrine cancers, hypocalcaemia, parathyroid hormone and vitamin D deficiency.

## Main text

### Malnutrition, overweight and obesity

Abnormal body weight and related metabolic disturbances are common issues for GD1 patients. Low appetite is one of the main symptoms of GD1, with an estimated incidence of 24.2% [7]. The percentage of underweight GD1 patients is estimated to be from 3 to 5% in adults [8]. Underweight seems to be more prevalent among untreated GD1 (5–9%), than in treated patients (2–4%) [8]. The prevalence of underweight in pre-ERT children and adolescents with GD1 is between 5 and 67%, depending on the age of diagnosis, severity of GD and country/nationality [8–12]. Unfortunately, there is limited data available on the development of malnutrition in GD1 paediatric patients treated with ERT. Oliviera et al. found that GD1 paediatric patients showed an initial malnutrition rate calculated using the Mora method of 26%, and after 5.3 years it increased to 48% [13]. Seventy seven percent of the patients were treated with ERT during the study. The response to the treatment is not reliably measurable because the treatment was not on a regular basis, due to its high costs [13]. The safety and effectiveness of SRT in patients aged from 2 to 17 is still being tested. Heitner et al. analysed the effectiveness of low doses of imiglucerase (±10 UI/kg every 2 weeks) used for at least 2 years in 9 GD1 paediatric patients from South Africa. An increase in weight was observed over time, with an average of 3.9 kg per year (95% confidence limits 3.60–4.24 kg/year) [14]. A precise, useful summary of weight and height changes during ERT treatment in GD children and adolescents is available in a review by Doneda et al. [15].

Although the prevalence of overweight in untreated patients with GD1 is lower than in healthy individuals, after long-term ERT therapy it becomes approx. 56%, which is similar to the general population [16, 17]. The reason might be attributed to the reduction of the resting energy expenditure (REE) due to the therapy, and the failure to adjust the calorie intake [16, 17]. REE is elevated by about 24% before the intervention, as a result of some metabolic disturbances [18]. GD1 patients have REE approximately 44% higher than predicted REE [19]. The weight changes in untreated GD subjects could be explained by the lower severity of the basic disease. Grigorescu Sido et al. observed a mean weight gain of 4.2 kg after 18 months of ERT in GD1 patients from Romania [20]. Giraldo et al. studied the Spanish population of GD1 patients at the average age of 44.8 ± 16.6 years. According to this study, 29% of the subjects were overweight or obese. The obesity and overweight were more common in patients treated with ERT or SRT (mean time on treatment was 10 years for all subjects) than in untreated GD1 patients. Splenectomised patients had a higher mean age (50.9 ± 13.8 years vs. 42.7 ± 17.1 years, *p* < 0.05) and were more often overweight or obese compared with non-splenectomised patients (28 vs. 21% for overweight and 8 vs. 6% for obesity) [8].

In summary, untreated GD1 patients appear to be at risk of malnutrition, especially in childhood. Adult, ERT-treated GD1 subjects are at a higher risk of overweight and obesity. The small sample size of most analysed groups of GD1 patients in the literature could explain the significant differences in anthropometric data between these studies (Table 1).

**Table 1:** Studies assessing nutritional status disorders in adult and paediatric GD1 patients

Author (year)	Study population	Main findings		
Barton et al., 1989 [19]	25 untreated GD1 patients, both children and adults	REE^a^ 44% higher than estimated REE		
Corssmit et al., 1995 [18]	7 untreated, adult GD1 patients	REE^a^ 24% greater than in age-, sex- and weight-matched healthy subjects		
Doneda et al., 2011 [21]	14 GD1 patients (12 on ERT, 2 untreated), both children and adults	BMR of patients 6.3% higher than expected REE. Observed BMR of patients on ERT 27.1% higher than in age-, sex- and BMI-matched controls. No difference between the BMR of subjects with or without ERT		
Giraldo et al., 2016 [8]	108 GD1 patients, 99 adults, 9 children 88% were receiving ERT or SRT	Prevalence of underweight (BMI < 18.5) - 3% in all GD1 patients - 2% in treated with ERT/SRT, 9% in untreated. Prevalence of overweight and obesity - 29% in all GD1 patients - 27% in treated with ERT/SRT, 46% in untreated. Higher incidence of overweight and obesity in splenectomised vs. non-splenectomised patients - 36 vs. 27%		
Grigorescu Sido et al., 2007 [20]	24 GD1 patients, both children and adult, evaluated before and during ERT for at least 18 months.	A mean weight gain of 4.3 kg during 18 months of ERT		
Heitner et al., 2004 [14]	9 paediatric GD1 patients, followed up for 2–10 years during ERT	An increase in weight during ERT was observed with an average weight gain of 3.9 kg per year		
Hollak et al., 1997 [17]	12 adult GD1 patients, evaluated before and after 6 months of ERT	Pre-ERT REE^a^ 25% greater than in age-, and weight-matched healthy subjects. Decrease of REE^a^ after 6 months of ERT from 129 to 120% of expected values. Appearance of increase in weight (mean 1.7 +/− 0.8 kg) after 6 months of ERT; mainly a significant increase in fat mass (mean 1.6 +/− 1.5 kg)		
Langeveld et al., 2008 [16]	42 adult GD1 patients (7 untreated, 35 on ERT		untreated	treated
		Initial overweight incidence	14%	16%
		Weight gain	3.8 kg or 2.2 kg/m^2^ during 8.2 year-follow up	6 kg or 2.4 kg/m^2^ during 11 years of ERT
		Final overweight incidence	57%	56%
		Data presented as median values		
		Weight changes in untreated GD1 subjects similar to the controls and to treated GD1 patients.		
Oliveira et al., 2002 [13]	13 paediatric GD1 patients, 77% treated with ERT	Pre-ERT malnutrition rate - 26% . Post-ERT (after a median therapy of 5.3 years) malnutrition rate increased to 48%		

*BMI* body mass index; *BMR* basal metabolic rate; *ERT* enzyme replacement therapy; *GD1* Gaucher disease type 1; *REE* resting energy expenditure; *SRT* substrate reduction therapy

^a^ determined by indirect calorimetry

### Increase in basal glucose production, insulin sensitivity, insulin resistance

An elevation of basal hepatic glucose production by about 30% is characteristic for ERT-naive GD1 [18]. This study indicated that an increase in endogenous glucose production is linked with more intense glucose disposal, and it confirmed that plasma glucose concentrations are at the same level as in healthy individuals [18]. The differences in post-absorptive glucose production in the liver are not accompanied by differences in plasma concentrations of glucoregulatory hormones, e.g. insulin. The mechanism cannot be clearly understood through common endocrine mechanisms. Corrsmit et al. postulated that changes in the non-endocrine intrahepatic regulatory system expressed as the interactions between macrophages, producing i.a. cytokines, adenosine, prostaglandins and hepatocytes, could alter glucose production [18]. Hepatic macrophages, like all macrophages in GD1, occur in a state of persistent activation without any alteration of hepatocyte functions [18].

Insulin resistance constitutes the major etiologic defect that defines metabolic syndrome. Insulin resistance is a metabolic key component of obesity and is a major factor in the aetiology of a number of diseases, including type 2 diabetes (DM II) and cardiovascular diseases [22–25]. Langeveld et al. described a 6% prevalence of insulin resistance in patients with ERT-treated GD1 [16]. However, the incidence of insulin resistance in GD is relatively low compared to studies of the general population [26]. A review by Fuller et al. discussed the altered sphingolipid metabolism seen in GD that leads to a significant decrease in insulin sensitivity. Therefore, GD is a good model to examine the role of sphingolipids in the development of insulin resistance [27]. Besides glucosylceramide storage, there are other elevated lipids in GD, including glycosphingolipid GM3 [28]. Ghauharali*-*van der Vlugt et al. suggested that the increase in GM3 storage has a prominent influence on the development of insulin resistance in patients with GD1 [29]. Studies show that the accumulation of GM3 results in a loss of the insulin receptors from lipid rafts [30, 31]. Independently, altered lipid raft composition in patients with GD causes disturbances in the Protein Kinase B (AKT) pathway. The physiological function of AKT in muscle involves glucose uptake and glycogen synthesis [32]. Activation of AKT1 in pancreatic islets results in an expansion of β-cell mass and an increase in insulin production [32]. The AKT pathway is essential for the inhibition of liver glucose production and the stimulation of lipid synthesis [29]. Cho et al. showed that mice with dysfunctional AKT2 developed insulin resistance, glucose intolerance and were susceptible to DM II [33].

Type M1 macrophages are known for secreting pro-inflammatory cytokines such as interleukin 6 (IL-6) and t*umour* necrosis factor α (TNFα). Increased concentrations of IL-6 and TNFα were observed in both GD and insulin resistance [34]. In addition, lipotoxicity in macrophages might be a link between GD and insulin resistance [27]. It appears that the activation of macrophages in GD, which leads to the development of systemic inflammation, could result in insulin resistance in GD [25, 27, 34]. More research is needed to determine the potential role of M1 macrophages in developing insulin resistance. Hypothetically, ERT could have a positive impact on insulin sensitivity and may prevent the development of DM II. Insulin resistance is also linked to systemic inflammation [25]. ERT removes the storage material from the cells and could decrease the production of GM3, for which glucosylceramide is a precursor. According to this mechanism, ERT could improve insulin sensitivity. On the other hand, ERT induces weight gain in a large number of patients, according to data provided in the section “Malnutrition, overweight and obesity”. Overweight is linked to the risk of insulin resistance development [8, 16, 17, 35]. It was found that pharmacological inhibition of glucosylceramide synthase has a positive impact on insulin resistance in cultured adipocytes from obese patients and rodents [36, 37]. ERT inhibits macrophage activation and reduces cytokine levels, resulting in an anti-inflammatory effect [27]. However, Langeveld et al. described the problem of peripheral insulin resistance also occurring during ERT. Insulin resistance could be linked to decreased glucosylceramide levels or transient growth of ceramide levels during ERT [16]. Ucar et al. showed that non-overweight GD1 patients treated with ERT also demonstrate insulin resistance [38]. In a study by Langeveld et al. before the initiation of ERT, none of the patients was diagnosed with DM II, but during ERT the prevalence of DM II increased significantly. DM II was diagnosed in four patients during ERT, increasing the pre-treatment incidence of diabetes from 0 to 8.2% after a median treatment time of 11 years. In the same study, no new cases of DM II were observed in the group of untreated patients during the follow-up [16]. In a prospective, controlled study of insulin resistance in GD 1 patients, baseline glucose parameters were comparable in GD1 subjects and controls and, after 3 years of ERT, the subjects were more insulin resistant vs. controls [39].

Further studies are needed to explain the mechanism and evolution of insulin resistance in both treated and untreated GD1 patients. The diagnostics and treatment of insulin resistance and DM II, both being possible long-term complications of GD1, are important general management goals in this disease and were adapted by Bennett et al., with minor modifications, from the Expert Consensus of the European Working Group on Gaucher Disease [40] (Table 2).

**Table 2:** Studies assessing carbohydrate metabolism disorders in adult GD1 patients

Author (year)	Study population	Main findings		
Corssmit et al., 1995 [18]	7 untreated, adult GD1 patients	Hepatil glucose production^a^ increased by 30% in GD patients vs. age-, sex- and weight-matched controls. Increased glucose production not related to increased plasma concentrations of glucose or glucoregulatory hormones.		
Ghauharali*-*van der Vlugt, 2008 [29]	40 untreated, adult GD1 patients	Elevated plasma GM3 concentrations in most GD1 subjects. GM3 plasma levels correlated with plasma chitotriosidase activity overall severity of disease and hepatomegaly.		
Hollak et al., 1997 [17]	7 GD1 adult patients, evaluated before and after 6 months of ERT	Initial hepatil glucose production^a^ increased by 23% in GD patients vs. age-, and weight-matched controls. No differences in plasma glucose concentrations in GD patients vs. controls. Persistent increase in glucose production after 6 months of ERT		
Langeveld et al., 2008 [16]	42 adult GD1 patients (7 untreated, 35 on ERT)	parameter	untreated	treated
		Fasting glucose Fasting insulin IR prevalence^b^ DM II prevalence	5.2 mmol/l 53 pmol/l 0% 0%	4.9 mmol/l 50 pmol/l 6% 8.2%
		Data presented as median values		
Langeveld et al., 2008 [35]	6 adult GD1 patients (3 untreated, 3 treated with ERT)	Peripheral insulin resistance in GD1 subjects compared with age-, sex- and BMI-matched controls. Comparable NIMGU at euglycaemia and hyperglycaemia in patients and control subjects. Lower IMGU in patients vs. controls. Tendency toward less effective suppression of lipolysis by cinsulin in patients vs. controls		
Ucar et al., 2009 [38]	14 non-overweight, adult GD1 patients on ERT	IR incidence^b^ 6.6%. IR in GD1 is not related to overweight. Higher basal insulin, glucose and insulin at 2 h after load and HOMA-IR in GD vs. healthy subjects		
Zimmermann et al., 2015 [39]	12 treatment-naive, non-overweight, adult GD1 patients; assessed before and every 6 months up to 3 years under ERT	No differences in baseline glucose parameters between GD1 subjects and age-, sex- and BMI-matched controls. Significant increase in HOMA-IR under ERT after 18 months (+ 61.2%). Higher levels of HOMA-IR, basal insulin and glucose after 3 years of ERT vs. controls		

*BMI* body mass index; *DM II* type 2 diabetes mellitus; *GD1* Gaucher disease type 1; *HOMA IR* homeostasis model assessment of insulin *resistance; IMGU* insulin-mediated glucose uptake; *IR* insulin resistance; *NIMGU* non-insulin-mediated glucose uptake

^a^ hepatic glucose production was measured by infusing radio-labelled 3-3H glucose

^b^ assessed when HOMA-IR > 4.65

### Lipid profile

GD1 appears to change lipoprotein concentrations. The influence of these changes on atherogenic processes is still under study. Serum concentrations of total cholesterol (TC), low-density lipoprotein cholesterol (LDL-C), and high-density lipoprotein cholesterol (HDL-C) are often low in untreated GD1 patients [41–43]. Low HDL cholesterol levels in GD1 do not lead to an increased risk of cardiovascular disease according to de Fost et al. [44]. Enhanced LDL-C/HDL-C ratios and decreased apolipoprotein (apo) A-I and B levels are common laboratory abnormalities at the time of diagnosis [41, 43] and plasma levels of apo E are typically increased [43]. The lipid profile also changes dynamically during ERT in previously untreated GD1 patients [43]. ERT results in a significant increase in HDL-C after 6 months (29.2 ± 5.7, *p* = 0.023), a decrease in LDL-C/HDL-C ratio after 30 months (2.5 ± 0.5, *p* = 0.039), a decrease in triglycerides (TG) after 18 months and an insignificant increase in LDL-C [41]. Although GD1 patients on ERT show a significant increase in HDL-C concentrations (+ 38%), persistently reduced HDL-C levels after 3 years of treatment have been reported as a potential risk factor of atherosclerotic changes [41, 43]. SRT with miglustat in previously untreated GD1 patients appears to increase HDL-C, TC and apoA-I and decrease TC/HDL-C ratios after 24 months [45]. In patients who switched from ERT to SRT, no changes were observed [45]. ERT and SRT appear to have beneficial effects on altered lipid profiles in GD1 patients [45].

Alterations in lipid profile correlate with disturbed ghrelin, leptin and adiponectin levels in GD1 patients. A direct correlation between adiponectin and ghrelin levels with HDL-C were found by Doneda et al.. Leptin levels were inversely proportional to LDL-C and directly proportional to triglyceride concentrations [46].

Genotype-phenotype correlations between *GBA* and *ABCG8* mutations and lipid profile were found. Significantly higher levels of TC and LDL-C are characteristic of patients with the GG genotype of D19H and CC genotype of T400K (*ABCG8* gene) [47]. More severe genotypes of *GBA* gene (N370S/84GG, N370S/L444P, N370S/IV2 + 1 and N370S/V394 L) are linked with significantly reduced HDL-C levels compared to less severe genotypes [48]. HDL-C concentration and LDL-C/HDL-C ratio are considered to be plausible indicators of the disease severity in ERT-treated GD1 subjects [41]. Stein et al. evaluated HDL-C as a possible biomarker of the disease severity [49]. HDL-C levels were found to be negatively correlated with the disease severity score index, liver and spleen volumes [49]. The degree of the association of GD1 severity and HDL-C levels was similar to that of classic GD1 marker - chitotriosidase [49].

An increased prevalence of gallstones (five times higher) was found in GD1 patients (38). Cholelithiasis occurs in 32–45.9% of the patients [47, 50]. The incidence of choleolithiasis increases with age and appears to be higher in young women and older men (in men: from 4.2% during their 20s to 71% at age > 70; in women: from 11.8% during their 20s to 60% at age > 70) [50]. High TC and LDL-C concentrations are risk factors for choleolithiasis [47]. Asplenic patients are at increased risk of choleolithiasis [50]. However, rapid implementation of ERT and avoidance of splenectomy may reduce the risk of gallstones [47, 50] (Table 3).

**Table 3:** Studies assessing lipid metabolism disorders in adult GD1 patients

Author (year)	Study population	Main findings
Alfonso et al., 2003 [43]	70 adult GD patients (54 on ERT, 16 without treatment)	Increase of HDL-c (+ 38%) and apo A-I (+ 18%) levels in ERT-treated patients. No significant effect of ERT on LDL-c and apo B levels. Decrease of plasma-apo E (− 32%) and HDL-apo E (− 26%) concentrations after ERT
de Fost et al., 2008 [44]	40 adult GD1 patients (34 on ERT, 2 on STR, 4 untreated)	Significantly lower levels of HDL-c and ApoA1 and higher TG levels in patients vs. controls. No differences in cIMT between patients and controls.
Puzo et al., 2010 [45]	26 adult GD1 patients treated with SRT for up to 36 months	Increase in plasma HDL-c and apoA-I in therapy-naive patients after SRT. Decrease in levels of TG, CRP concentrations, and TC/HDL-c ratios after 24 months of SRT. No changes in HDL-c and apoA-I, or in the TC/HDL-c ratio in patients switched from ERT to SRT
Stein et al., 2011 [49]	278 adult, GD1 patients, evaluated before and during ERT.	Significantly lower HDL-c in untreated GD1 subjects. A negative correlation between HDL-c chitotriosidase level, spleen and liver volume, GD severity score index. A rise of initially low HDL-c towards normal after ERT
Zimmermann et al., 2013 [41]	12 treatment-naive adult GD1 patients, evaluated before and after 3 years of ERT.	Pre-ERT: decreased HDL-c concentrations, increased LDL/HDL ratios (3.1 ± 0.7). During ERT: Increase of HDL-c after 6 months of ERT. Decrease of LDL/HDL ratio after 30 months of ERT. Decrease of TG after 18 months of ERT. Small dense LDL concentration increased constantly and was comparable to controls. After 3 years of ERT: reduced HDL-C concentrations, however, mean concentrations significantly improved

*apo B apolipoprotein B*; *apoA1* apolipoprotein A; *cIMT* Carotid intima-media thickness; *CRP* C-reactive protein; *ERT* enzyme replacement therapy; *GD1* Gaucher disease type 1; *HDL-c* high-density lipoprotein cholesterol; *LDL* low-density lipoprotein cholesterol; *SRT* substrate reduction therapy; *TC* total cholesterol; *TG* triglycerides

### Adiponectin

Adiponectin is a secretory protein which is produced by adipose tissue cells. Adiponectin enhances fatty acid biosynthesis and represses gluconeogenesis in the liver [51] and also improves glucose uptake by skeletal muscles [52]. Adiponectin increases insulin sensitivity and has antioxidant, anti-inflammatory and anti-atherosclerotic effects [53]. Hypoadiponectinaemia is usually linked to obesity, insulin resistance and DM II [54–56]. Langeveld et al. revealed a prominent low serum level of adiponectin in untreated patients with GD1 [57]. As mentioned earlier, a decreased level of adiponectin is often accompanied by obesity in healthy individuals, but not in the case of patients with GD1 [54]. Langeveld et al. showed that no relationship between adiponectin level and body mass index (BMI) exists in untreated GD1 patients [57]. They also observed that the negative correlation between adiponectin levels and BMI reappeared after long-term treatment with ERT. Furthermore, no clear relation between the level of adiponectin and the amount of Gaucher cells in liver, spleen and bone marrow was found [57]. Adiponectin levels do not depend on the accumulation of Gaucher’s cells. The study also showed that the regression in organomegaly is not accompanied by an increase in adiponectin levels. Initially, the administration of ERT produces a moderate increase in adiponectin. It is known that treatment in GD1 removes only a part of the altered cells without lowering the systemic inflammation entirely. The remaining low-grade systemic inflammation, despite treatment, may prevent the restoration of adiponectin levels, which might be completely suppressed in the long run [57].

Doneda et al. found, opposite to the results of Langeveld et al. [46], that there is no difference between the median levels of adiponectin in GD1 patients treated with ERT and controls. Moreover, they indicated that GD1 patients with splenomegaly have lower levels of adiponectin when compared to GD1 patients without splenomegaly. Adiponectin concentrations were inversely proportional to the BMI, waist circumference and TG and directly proportional to HDL-C [46]. These results come from patients with GD1, all of whom were on ERT for at least 6 months and their BMI was higher than in the Lagneveld et al. study. Additionally, it should also be noted that blood samples were taken before initiating the therapy in the study by Langeveld et al. [18, 57]. ERT seems to have a significant effect on adiponectin concentrations in GD1 patients.

### Leptin and ghrelin

Leptin and ghrelin hormones have an impact on appetite control and energy balance. Leptin is a well-known long-term regulator of energy balance by food intake suppression and weight loss [58]. The high-speed action of ghrelin stimulates appetite and food intake as well as growth hormone (GH) secretion [59].

The average concentrations of leptin and ghrelin are comparable in treated GD1 patients and in controls [46]. Doneda et al. found that patients with metabolic syndrome and GD1 on ERT had significantly higher levels of leptin, BMI, waist circumference, TG and insulin compared to GD1 cases without metabolic syndrome [46]. A negative correlation between BMI, waist circumference, TG levels and ghrelin concentrations was observed in GD1 patients. However, serum ghrelin levels were positively correlated with HDL-C. Leptin showed a positive correlation with BMI, waist circumference, TG, insulin and a negative correlation with LDL-C. A strong association between levels of leptin, insulin and homeostatic model assessment of insulin resistance index (HOMA-IR) was also found in GD1 subjects. This association was not present in the controls [46].

Agilli et al. drew attention to several points that needed to be clarified in the study by Doneda et al. [60], particularly the strong correlation reported between leptin levels and HOMA-IR and the probable usefulness of this result to detect the first signs of insulin resistance in patients with GD1. Agilli et al. emphasized the importance of specifying the menstrual status of women involved in the study and the possible impact of medications and dietary supplements [60]. Riad-Gabriel et al. revealed significant fluctuations of plasma levels of leptin during the menstrual cycle which depend on progesterone levels [61]. It also appears to be important to specify if the participants were using some types of antidepressants, antipsychotics, glucocorticoids, statins, antidiabetic and/or antihypertensive drugs, which may affect plasma leptin levels [62]. Furthermore, dietary supplements may also significantly influence leptin concentrations [63]. Therefore, these factors should be considered in order to draw reliable conclusions and the role of leptin level disturbances in GD1 should be clarified in further studies (Table 4).

**Table 4:** Studies assessing adopinectin, leptin and grelin in adult GD1 patients

Author (year)	Study population	Main findings
Doneda et al., 2015 [46]	15 adult, ERT-treated GD1 patients	No differences in median levels of ghrelin, leptin and adiponectin between patients and controls. Lower adiponectin concentrations in patients with splenomegaly vs. subject without splenomegaly. Inverse correlation between both ghrelin and adiponectin levels and BMI, waist circumference and TG. Positive correlation between both ghrelin and adiponectin levels and HDL-c. Inverse correlation between leptin and LDL-c and a direct correlation between leptin and BMI, waist circumference, enzyme dosage, TG, insulin, and HOMA-IR.
Langeveld et al., 2007 [57]	28 adult GD1 patients (26 on ERT, 2 on SRT), assessed before and after several years of therapy	Significantly lower serum adiponectin concentrations in untreated patients compared with healthy BMI-matched controls. Post-ERT adiponectin levels increased significantly, but remained lower vs. those of BMI-matched controls No correlation between adiponectin level and BMI in untreated GD1 patients

*BMI* body mass index; *ERT* enzyme replacement therapy; *GD1* Gaucher disease type 1; *HDL-c* high-density lipoprotein cholesterol; *LDL* low-density lipoprotein cholesterol; *SRT* substrate reduction therapy; *TG* triglycerides

### Growth hormone deficiency (GHD), delayed puberty and gynaecologic symptoms

Delayed puberty and growth retardation are believed to be linked to the disease itself and its severity [64]. Growth retardation usually occurs between the ages of 2–5 years [10, 15]. Untreated GD1 requires a high-calorie diet, which may eventually lead to growth inhibition [65]. The coexistence of hypermetabolism with healthy thyroid functioning could result in growth hormone deficiency (GHD) in GD1 according to Langeveled et al. [66]. Disturbances in the regulation of insulin and metabolism of glucose, free fatty acids and amino acids may disturb the function of GH/IGF-1 axis [67]. Kaplan et al. assessed 887 untreated GD1 paediatric patients in the context of growth retardation. Linear growth deceleration was observed in 34% patients at the time of diagnosis [68]. The median height was below the population mean, especially in the case of earlier onset of clinical signs and symptoms [68]. Rite et al. found the growth rate of 19 Spanish GD1 patients was associated with the IGF-1 concentration before and after ERT [69]. Pre-ERT levels of IGF-I and IGF binding protein 3 (IGFBP-3) were low and directly proportional to the standard height deviation. A significant increase in IGF-1 and IGFBP-3 concentrations and their normalization was achieved after 1 year of ERT [69]. ERT has a remedial effect on height [15]. Zevin et al. studied 34 children and adolescents with GD1 [10] and found that growth deceleration was observed in 30% of the patients and the severity of growth retardation was correlated with the genotypes [10]. Patients carrying the p.N370S/84GG and L444P/L444P genotype have more severe disease and degree of growth retardation. A corrective effect of ERT was observed in all of the nine patients who finished a twelve-month treatment course [10]. ERT seems to improve the percentile and/or z-score of the height by 50–80% in different studies [10–13, 70]. The therapeutic goal of ERT is to normalize growth and reach the peak of bone acquisition within 3 years of treatment onset [71]. Noticeable improvements in GHD after ERT may suggest that the GHD is related to the metabolic disturbances rather than to primary endocrine pathology [72].

Other researchers support the view that the mean final Height Standard Deviation Score (HSDS) is not impacted by ERT [73]. Mendelsohn et al. showed that there is no difference in reaching a target height between treated and non-treated GD1 patients. Untreated children were significantly less affected by the enzyme deficiency than those who started ERT treatment early [73]. Mendelsohn et al. also observed that the final height was decreased in boys but not in girls, regardless of ERT, without a known reason [73]. Kauli et al. suggests that the recovery from growth retardation happens at puberty, independent of ERT treatment in prepubertal GD1 patients [65]. Delayed puberty, which often occurs in patients with GD1, can have an important impact on final height. Due to a longer time of growth, GD1 children are expected to reach a normal final height in their adulthood [73]. However, ERT seems to have a noticeable effect on the improvement of growth retardation in prepubertal patients [73]. This, in turn, has an evidently positive impact on the psychological and social development of children suffering from GD1. Drelichman et al. evaluated the clinical effects of interrupted ERT treatment in five children with GD1 [12]. Before ERT initiation, four of them (80%) suffered from growth retardation. All patients underwent a successful normalization of linear growth after 1 to 7 years of ERT. Growth retardation was observed in 3 of 5 patients (60%) after 15 to 36 months of ERT interruption [12]. Growth deceleration could be a severe consequence of a therapy pause and, as a result, ERT interruptions should be avoided in children.

Delayed puberty is a significant medical problem and it appears more often with severe manifestation in untreated GD1 patients. Moreover, as mentioned earlier, delayed puberty could exacerbate the growth retardation of children suffering from GD1. There are insufficient studies on delayed puberty in GD1 patients. However, they show that delayed puberty occurs in females as frequently as in males [65]. The age at menarche is significantly delayed in girls suffering from GD1 [73]. Delayed puberty may appear in two-thirds of GD1 patients without any further problems with infertility [74]. Granovsky-Grisaru et al. observed a single case of hypogonadotropic hypogonadism, within the group of 53 females [74]. Kauli et al. did not observe any positive effect of splenectomy (partial or total) at the onset of puberty [65]. A positive effect of ERT on the onset of puberty and QoL of GD1 patients was observed [65]. However, Kali and Zaizov et al. observed a delayed spontaneous catch-up of reaching the predicted height and complete sexual maturity, even in the case of a severe form of GD1 independent from ERT [65].

To sum up, the mechanism of GHD and growth retardation in patients with GD1 is a complex issue, which is not fully understood. Further research is needed to explain the problem of GHD and delayed puberty in GD1 (Table 5).

**Table 5:** Studies assessing growth retardation and delayed puberty in adult and paediatric GD1 patients

Author (year)	Study population	Main findings
Bembi et al., 1994 [11]	9 paediatric GD1and GD3 patients, assessed before and after at least 12 months of ERT	Initial growth retardation rate: 67%. A growth recovery in all children with initial growth retardation after 12–24 months of ERT
Drelichman et al., 2007 [12]	32 paediatric GD1 patients, including 5 with history of ERT interruption	Initial growth retardation rate: 80%. 100% growth recovery rate after 1 to 7 years of ERT. 60% of children experienced growth retardation after ERT interruption
Granovsky-Grisaru et al., 1995 [74]	53 GD female patients	Puberty delay in two thirds of the patients without later infertility. Growth retardation rate 2.8%
Ida et al., 1998 [9]	Untreated GD1 patients, 23 children and 12 adults	Significant exacerbation/deterioration of mean relative height and weight during 5-year follow-up.
Kaplan et al., 2006 [68]	887 untreated GD1 paediatric patients	34% of incidence of linear growth deceleration at the time of diagnosis. Decreased median height compared to *population standards in all age groups*
Kauli et al., 2000 [65]	57 paediatric GD1 patients, 63% on ERT	Growth retardation at age 3–5 years in 53% of subjects observed since early childhood while untreated. 59.6% prevalence of delayed puberty, linked with severity of the disease. All patients, both treated and untreated, with growth and pubertal delay caught up full sexual development. 83.3% of all subject achieved a predicted final height. A positive effect of splenectomy only on growth, not on puberty delay.
Mendelsohn et al., 2018 [73]	41 GD1 adult patients, both treated and untreated	Mean HSDS in the patients − 0.22. No difference in achieving a target height between treated and non-treated subjects. Noticeable effect of ERT on the improvement of growth retardation in prepubertal patient. Decrease in final height in boys but not in girls regardless of ERT. Delayed age at menarche in girls
Rite et al., 2002 [69]	22 GD1 paediatric patients, evaluated before and after ERT	Low levels of IGF and its binding proteins in treatment-naive patients. Increase of IGF-1 after 12 ± 6.8 months of ERT. IGF-I deficiency is linked with the disease severity.
Zevin et al., 1993 [10]	34 paediatric pre-ERT GD1 patients; 9 patients assessed after at least 12 months of therapy	Growth retardation at presentation: 26% on or *below* the *3rd percentile* for weight, and 30% so in height. Growth retardation mostly in the youngest age group (2–5 years). 43% of patients with initial growth retardation increased their height percentile, 57% increased their weight percentile during ERT

*ERT* enzyme replacement therapy; *GD1* Gaucher disease type 1; *HDSD* height standard deviation score

### Thyroid hormone status

Limited data are available on thyroid disorders in GD1. Hypermetabolic state and related changes in body composition, such as lower muscle and fat mass, are characteristic of both untreated GD1 and hyperthyroidism [17–19, 21, 75]. Increased energy metabolism in indirect calorimetry measurements was proven in several studies on GD [17–19, 21]. Excluding hyperthyroidism is crucial in patients with a highly expressed hypermetabolic state with sweating, diarrhoea malnutrition and/or menstruation disturbances (in women). Appropriate treatment should alleviate the hypermetabolic state in both GD1 and hyperthyroidism.

No correlation between thyroid hormone concentrations and indicators of hypermetabolism was observed in the Langeveld et al. study [66] in which thyroid hormone levels were measured in 22 GD1 adult patients prior to and during ERT. In 12 cases, REE were measured and correlated with thyroid hormone level. 17/22 (77%) patients had normal thyroid hormone levels at baseline. There was no association between serum levels of 3,3′,5-triiodo-l-thyronine (T3), free thyroxine (fT4), 3,3′,5′-triiodo-l-thyronine (rT3) and baseline REE (kcal/kg per 24 h). Long-term ERT caused a decrease in serum fT4 levels. A decline in REE was noted after several months of therapy. However, the decrease in serum levels of fT4 and T (3) did not correlate with the alteration in REE. In addition, no cases of non-thyroidal illness syndrome (NTIS) were noted [66]. No reliable data on thyroid nodules, thyroid autoimmune pathology, hypothyroidism or hyperthyroidism in GD1 are available in the literature (Table 6).

**Table 6:** Studies assessing thyroid hormone status and thyroid cancer in adult GD1 patients

Author (year)	Study population	Main findings
Langeveld et al., 2007 [66]	22 adult GD1 patients, assessed before and during ERT	Normal thyroid hormone levels in 77% patients at baseline.
		No correlations between serum levels of: 3,3′,5-triiodo-l-thyronine (T3), free thyroxine (fT4), 3,3′,5′-triiodo-l-thyronine (rT3) and baseline REE (kcal/kg per 24 h).
		A decrease in fT4 and REE during ERT. No correlation between decrease in fT4 and T(3) and changes in REE.
Lo et al., 2010 [76]	403 adult GD1 patients, treated and untreated	Two cases of papillary thyroid carcinoma concomitant with haematological malignancies in splenectomised subjects.

*ERT* enzyme replacement therapy; *fT4* free thyroxine, *GD1* Gaucher disease type 1; *REE* resting energy expenditure; *rT3–3,3′,5′* triiodo-l-thyronine; *SRT* substrate reduction therapy

### Calcium homeostasis and bones in GD1

GD1 patients are in a high-risk group of osteopenia, osteoporosis and fractures [77, 78]. The skeletal system, one of main organ systems affected in GD1, is under the influence of multiple hormones. A number of new substances with hormonal activity, such as osteocalcin, bone-specific alkaline phosphatase, vitamin D, parathyroid hormone and osteopontin have been studied in GD1 [79, 80]. Here, we discuss the aspect of calcium homeostasis and the issues of parathyroid hormone and vitamin D disturbances in GD1.

### Hypocalcaemia and hypoparathyroidism

Calcium homeostasis is maintained by parathyroid hormone (PTH) and vitamin D. Approximately 33% of GD patients treated with ERT experience temporary hypocalcaemia 10 to 12 days after the start of the treatment [11]. Transient hypocalcaemia accompanied by normocalcuria could be linked with an increase in calcium bone deposition and an improvement in bone metabolic disease [11]. The timing of calcium measurements is important to help in preventing temporary hypocalcaemia during ERT. Oral administration of 500–1000 mg of calcium and 400–800 IU of vitamin D daily should be recommended to prevent hypocalcaemia [11].

The major causes of hypocalcaemia in the general population are vitamin D deficiency, hypomagnesaemia and hypoparathyroidism. Gaucher disease associated with hypoparathyroidism is a condition described in a case report by Sultan et al. In this study a 10-year-old patient diagnosed with GD1 and R359*/N370S genotype presented with hypocalcemic convulsions and hypoparathyroidism [81]. The presence of both diseases in one individual is more likely to be a coincidence. Further studies are needed to observe if hypoparathyroidism in GD1 patients is a comorbidity or part of the clinical picture of GD1.

An interesting case report of a GD1 patient with lung and cardiac involvement with pericardial and valvular calcifications and hypocalcaemia was described by Tofolean et al. [82]. Unfortunately, no data regarding the genetic testing of the patient was provided.

It is essential to exclude hypoparathyroidism and maintain the correct level of calcium, magnesium and vitamin D in GD1 patients through proper diet and/or dietary supplements.

### Vitamin D deficiency

Vitamin D deficiency is very common among the general population. Serum 25-hydroxyvitamin D (25[OH]D) levels are the gold standard parameter to evaluate vitamin D deficiency [83, 84]. There is some disagreement among experts on appropriate blood levels of vitamin D, which makes it difficult to clearly define a deficiency*.* As a result, many experts consider a broad definition of vitamin D deficiency using a cut-off level of 25(OH) D of less than 50 nmol/L (20 ng/mL) [85, 86]. A 25(OH) D level of 50 to 125 nmol/l (20 to 50 ng/ml) is considered adequate for healthy individuals [84, 86]. Over 70% of the general population and around 83% of GD1 patients are vitamin D deficient [87].

Mikosch et al. found a total mean of 25(OH) D level of 58.2 ± 30.3 nmol/L (23.28 ± 12.12 ng/ml) in 60 GD1 patients from England, which is clearly below the threshold of deficiency [87]. GD1 patients have a higher prevalence of moderate-to-severe vitamin D insufficiency compared to the healthy population of 45-year-old subjects living in southern England [87, 88]. A seasonal variation in the 25(OH) D values was detected in the GD1 population. Lower levels of 25(OH) D observed during the winter and spring showed significant correlations with indicators of osteopenia and osteoporosis - T-scores and Z-scores of the lumbar spine and hip [87]. Parisi et al. studied a small group of nine young GD1 patients treated with ERT and found that all patients presented with hypovitaminosis D, defined as 25(OH) D levels < 75 nmol/L (< 30 ng/ml) [89]. Hypovitaminosis D in GD1 may result from: poor dietary intake, intestinal malabsorption, diminished exposure to ultraviolet light, and/or poor skin production or decreased hepatic production of calcidiol. Increased bone mineral density (BMD) has been confirmed in GD patients treated with ERT and receiving interrupted vitamin D supplementation [90]. There is one negative study on the efficacy of small doses of calcitriol (1,25-dihydroxyvitamin D3; 0.25–3.0 μg/day) alongside ERT on the improvement of bone density in splenectomised GD1 subjects. The doses of vitamin D administered in that study were too small to reliably evaluate the vitamin D effect on BMD [91].

According to the “Management goals for type 1 Gaucher disease: An expert consensus document from the European working group on Gaucher disease”, no consensus was achieved for statements on vitamin D deficiency. The diagnosis and treatment of hypovitaminosis D was regarded as good clinical practice rather than a management goal for GD1 [92]. Measurements of serum calcium and vitamin D concentrations are highly recommended in all GD1 patients. Vitamin D supplementation is necessary when the 25(OH) D level is less than 75 nmol/L (30 ng/ml) [93, 94]. GD1 patients should take an effective regimen of vitamin D and oral calcium supplementation in the case of insufficient food intake of calcium. Cholecalciferol, in a dose of minimum 800–1000 mg (IU/d) per day, seems to be the preferred preparation for patients with GD1 [95]. However, to achieve a blood concentration of 25(OH) D above 30–40 ng/ml, GD1 patients may need at least 1500–2000 mg (IU/d) of vitamin D (85). Doses of vitamin D supplements should be individualized depending on age, body weight, sun exposure, dietary habits, lifestyle and concomitant diseases [95]. According to Hughes et al., adequate calcium and vitamin D prevention and therapy in GD should be conducted according to the local guidelines for vitamin D deficiency, both in children and adults [96]. An appropriate dose of vitamin D based on the monitoring of 25(OH) D concentrations and ascertained by recommended laboratory assays would optimize the treatment of hypovitaminosis D [95]. Vitamin D supplements should be taken with a healthy high-fat meal, not together with high-fibre food or laxatives [97]. The efficacy of continuous combined therapy with vitamin K2 and vitamin D3 has not yet been proven [95, 97]. In conclusion, GD1 subjects require an individualized long-term vitamin D and calcium therapy for bone health [87].

Studies on *vitamin D receptor (VDR)* gene polymorphisms have become popular recently in the general population and in specific groups of patients, including GD1 patients. The AA genotype of the c.1024 + 283G > A (rs1544410) gene variant in the *VDR* gene seems to be a risk factor for low BMD, osteoporosis and pathological fracture in GD1 patients [98]. *VDR* Bsml polymorphism was associated with skeletal involvement, including osteonecrosis and/or pathological fractures, in GD1 subjects [99]. Lieblich et al. found that there was an association of single-nucleotide polymorphisms (SNPs) in the *VDR* gene (ApaI aa genotype) and malignancies in GD patients [100]. More studies are required to determine the potential preventive effect of vitamin D supplementation against bone diseases, neoplasm and other concomitant diseases in GD patients (Table 7).

**Table 7:** Calcium homeostasis and vitamin D in adult and paediatric GD patients

Author (year)	Study population	Main findings
Bembi et al., 1994 [11]	12 GD1 and GD3 patients, including 9 children, assessed before and after at least 12 months of ERT	A temporary hypocalcaemia 10–12 days after the start of the treatment in 33% patients.
Mikosch et al., 2009 [87]	60 GD1 adult patients, most on ERT or SRT	83.0% of patients with insufficient vitamin D level (≤80 nmol/L). 17.0%, of patients with sufficient vitamin D level (> 80 nmol/L). Presence of a significant seasonal variation of 25(OH) D values A significant correlation between the 25(OH) D level during the season December–May and T-scores and Z-scores of the lumbar spine and hip
Parisi et al., 2008 [89]	9 GD1 young adult patients on ERT	Hypovitaminosis D (25(OH) D levels < 75 nmol/L) in 100% patients.
Schiffmann et al., 2002 [91]	29 splenectomised GD patients	No significant effect of small doses of calcitriol (1,25-dihydroxyvitamin D3; 0.25–3.0 μg/day) alongside ERT on the improvement of bone density .
Zimmermann et al., 2018 [98]	50 adult GD1 patients	The AA genotype (c.1024 + 283G > A gene variant; VDR gene) - a risk factor for low BMD, osteoporosis and pathological fractures.

*BMD* bone mineral density; *ERT* enzyme replacement therapy; *GD1* Gaucher disease type 1; *SRT* substrate reduction therapy; *VDR* vitamin D receptor

## Summary

A number of studies have suggested a link with different metabolic changes, although the clinical significance of this is questionable. The heterogeneity of the patients’ clinical profile and the low number of subjects were among the limitations of the studies that made it challenging to confirm whether the obtained findings were intrinsic to GD as a detrimental or a compensatory process. What is more, some changes may in fact be co-morbidities. Regarding the liver, it may be dysfunctional, although the rare occurrence of liver failure is rather a consequence of conditions such as, e.g. hepatitis, autoimmune diseases or infections. Finally, it is necessary to focus on other disease processes and mechanisms when individuals with a diagnosis of GD do not follow a typical course of the disease or show characteristics, which are inconsistent with the key features of reticuloendothelial involvement.

## Conclusions

Metabolic and hormonal diseases are widespread in the world population. Gaucher disease appears to facilitate the development of some of the discussed diseases, especially disorders of nutrition and glucose metabolism. Many metabolic and hormonal diseases result in pathological changes, often with the most severe effects on the course of the underlying disease and patient’s quality of life. GD1 patients are at increased risk of peripheral insulin resistance and characteristic lipid alterations. An increased focus on detecting hormonal and metabolic disturbances, especially nutritional status disorders, insulin resistance and lipid alterations, is strongly recommended to optimize the healthcare therapy in GD1 patients. Effective ERT treatment seems to have positive effects on most metabolic and hormonal diseases, but could promote a tendency to gain weight. The existence of a casual relationship and confirmed, reasonable pathophysiological mechanism, is hard to prove because of a low incidence of GD in the population and a relatively high incidence of metabolic and hormonal disorders, such as obesity or insulin resistance, in the general population. It is necessary to conduct further longitudinal studies on larger GD1 patient groups to monitor the risk of metabolic and hormonal disorders, especially with the emergence of SRT and the transition of patients to it from ERT.

## Data Availability

The datasets used and/or analysed during the current study are available from the corresponding author upon reasonable request.

## Abbreviations

25(OH)D: Serum 25-hydroxyvitamin D
AKT: Protein Kinase B
AMPK: 5’AMP-activated protein kinase
apo: Apolipoprotein
BMD: Bone mineral density
BMI: Body mass index
DM II: Type 2 diabetes
ERT: Enzyme replacement therapy
GD: Gaucher disease
GD1: GD type 1
GH: Growth hormone
GHD: Growth hormone deficiency
GM3: Glycosphingolipid GM3
GR: Growth retardation
HDL-C: High-density lipoprotein cholesterol
HMW: High–molecular weight
HOMA-IR: Homeostatic model assessment of insulin resistance index
IGF-1: Insulin growth factor-1
IGFBP-3: IGF binding protein 3
IL-6: Interleukin 6
LDL: Low-density lipoprotein
NTIS: Nonthyroidal illness syndrome
PTC: Papillary thyroid cancer
PTH: Parathyroid hormone
QoL: Quality of life
REE: Resting energy expenditure
SNP: Single-nucleotide polymorphism
SRT: Substrate reduction therapy
TC: Total cholesterol
TG: Triglycerides
TNFα: T*umour* necrosis factor α
VDR: Vitamin D receptor
